# A study evaluating the safety of laparoscopic radical operation for colorectal cancer

**DOI:** 10.4103/0972-9941.15243

**Published:** 2005-03

**Authors:** Min-Hua Zheng, Ai-Guo Lu, Bo Feng, Yan-Yan Hu, Jian-Wen Li, Ming-Liang Wang, Feng Dong, Jing-Li Cai, Yu Jiang

**Affiliations:** Department of Surgery, Ruijin Hospital affiliated of Shanghai Second medical University, Shanghai Minimally Invasive Surgery Clinical Center, Shanghai (200025), China

**Keywords:** Laparoscopy, colorectal tumour, radicality

## Abstract

**Aim:**

This study aimed to assess the safety and feasibility of laparoscopic curative resection for colorectal cancer through the clinical practice and basic research.

**Material and Methods:**

From September 2001 to September 2002, 47 patients with colorectal cancer were treated using laparoscopic approach, compared with 113 patients underwent traditional operation. The length of intestinal segment excised, size of tumour, clearance of lymph nodes, local recurrence and distant metastasis rate during the period of follow-up in both groups were compared. The other part of the study involved the detection of exfoliated tumour cells in the peritoneal washing before and after surgery; flushing of the instruments was performed in both groups and the results compared. For the laparoscopic cases, the filtrated liquid of CO_2_ pneumoperitoneum was also checked for tumour cells.

**Results:**

No significant differences existed in tumour size, operative site and manner between the two groups. The exfoliated tumour cell was not detected in the CO_2_ filtrated liquid. Between both groups there was no difference in the incidence of exfoliated tumour cells in peritoneal washing before and after surgery as well as in the fluid used for flushing the instruments. The total number of lymph nodes harvested was 13.71±9.57 for the laparoscopic group and 12.10±9.74 for the traditional procedure. Similar length of colon was excised in both groups; this was (19.38±7.47) cm in the laparoscopic and (18.60±8.40) cm in the traditional groups. The distal margins of resection for rectal cancer were (4.19±2.52) cm and (4.16±2.00) cm respectively. The local recurrence rate was 2.13% (1/47) and 1.77% (2/113) with the distant metastasis rate 6.38% (3/47) and 6.19% (7/113) respectively. Both the statistics were comparable between the laparoscopic and traditional surgery for the colorectal cancer.

**Conclusion:**

Laparoscopic curative resection for colorectal cancer can be performed safely and effectively. In the treatment of colorectal malignancy, laparoscopic resection can achieve similar radicalilty as compared to the traditional laparotomy.

## INTRODUCTION

Since the initial successful application of laparoscopic technique to resection of the colon by Jacobs and Fowler, this approach has been used fairly widely by several groups. With the maturity of surgical skills, refinements in instrumentation and availability of newer energy sources laparoscopic colorectal resections have become more successful and are associated with fewer complications. Despite this there some controversies still exist for the use of laparoscopic technique in the treatment of malignant tumour. The debated issues include the oncologic feasibility and safety and the likelihood of the port-site metastasis. This non-randomized, comparative study between laparoscopic surgery and traditional open procedure focussed on the problem of the safety of laparoscopic colorectal resection for malignancy.

## MATERIAL AND METHODS

### Patient Selection

One hundred and seventy eight patients with colorectal cancer operated between September 2001 and September 2002 by laparoscopic approach or open surgery were included in the study. The inclusion criteria were: 1) age: from 18 to 75 years old; 2) curative resection: 18 cases that were Duke’s D stage were excluded; thus 160 patients were analysed in this study. Among them, 47 patients underwent the laparoscopic colorectal resection (LCR) and 113 cases underwent open resection (OR). The type of operation was elected and determined by the patients themselves. Between the two groups, there were no significant differences in age, tumour location or postoperative staging (Table 1). The operations performed included right hemicolectomy, left hemicolectomy, transverse colectomy, sigmoid colectomy, low anterior resection and abdominoperineal resection. All operations were performed by an experienced surgeon specialized in laparoscopy and open surgery respectively.

**Table 1:** Demographic data of the laparoscopic group (Lap) and the open group (Open)

		Lap (n=47)	Open (n=113)	*P*
Tumour Location Number (%)	Caecum, ascending colon	7 (14.9%)	19 (16.8%)	*P*=0.21
	Transverse colon	3 (6.3%)	4 (3.5%)	
	Descending colon	1 (2.1%)	6 (5.3%)	
	Sigmoid colon	4 (8.5%)	24 (21.2%)	
	Rectum	32 (68.1%)	60 (53.1%)	
Duke’s stageNumber (%)	A	10 (21.3%)	36 (31.9%)	*P*=0.15
	B	21 (44.7%)	33 (29.2%)	
	C	16 (34.0%)	44 (38.9%)	
Age (yrs)	59.85±12.15	61.00±12.72	*P*=0.60	
Previous operation Number	15	35	*P*=0.91	

### Principles and technique of operation

Laparoscopic as well as open operations conformed to the oncological principles of surgery for colorectal cancer: avoidance of excessive tumour handling, protection of incision and adequate lymphatic clearance. For the malignant tumours of rectum, the procedure must incorporate a total mesorectal resection (TME). The laparoscopic group included laparoscopically-assisted colorectal resection and hand-assisted laparoscopic operations.

### Collection of CO_2_ filtrated fluid

In the laparoscopic group, pneumoperitoneum was established first and maintained at an intraperitoneal pressure of 15 mmHg. After 4 to 5 trocars were inserted, one of the side ports on the trocars was opened and the CO_2_ filtrated through 100 ml saline in a hermetic container. This fluid was then collected for analysis.

### Collection of the peritoneal washing

In both groups, samples for cytology were collected twice: firstly, immediately after the opening of peritoneal cavity and at time just before the abdominal closure. Upon opening the peritoneal cavity at laparotomy or after insertion of trocars, 100 ml to 200 ml saline was instilled and the fluid was aspirated; this served as a preoperative sample. At the end of operation the wound and instruments were rinsed with 500 ml~1000 ml saline and 100 ml irrigation fluid was collected in the same manner as the postoperative sample of peritoneal washing and the instruments flushing fluid.

### Examination of the exfoliated tumour cells

Cytological examination of the fluid samples collected was performed after the process of centrifugation, filtration, sedimentation, smearing, making of cell blocks and hematoxylin and eosin staining. Microscopic examination was performed to detect exfoliated cancer cells, and the positivity between the two groups was compared.

### Comparison of oncology results

A single pathologist blinded to the method of resection (LCR or OR) examined the specimens. The diameter of the tumour, length of the distal margin, length of the bowel excised, and the number of lymph nodes harvested were measured. Depending on the anatomic location, the lymph nodes were classified as 3 stations: epicolic and paracolic lymph nodes, intermediate lymph nodes, the principle lymph nodes (at the origins of the superior and inferior mesenteric vessels or colic artery).

### Follow up

All patients completed the follow-up. They all had a clinical examination, CEA and CA199 estimation and the chest x-ray. In addition, the ultrasonography of the liver, computerised tomography of the abdomen and colonoscopy were undertaken as needed.

### Statistical analysis

SPSS 10.0 software package was used for statistical analysis. Student *t*-test and Chi-square test were used to compare the categorical and parametric data respectively. P < 0.05 was considered statistically significant. All data were expressed as mean±standard deviation (SD).

## RESULTS

Both the groups were well matched for age, location and size of tumour and Duke’s stage (*P* > 0.05) (Table 1). In the laparoscopic group, 45 cases were completed totally laparoscopically by a hand-assisted laparoscopic operation. Two cases were converted to open procedure due to the dense adhesions.

In the LCR group, no tumour cells were detected in the CO_2_ filtrated fluid. The pre- and postoperative peritoneal fluid cytology was positive in 3/47 (6.38%) in LCR group and 8/113 (7.07%) in the OR group. The incidence of negative preoperative peritoneal cytology with a positive postoperative cytology was 2/47 (4.26%) in the LCR group and 5/113 (4.42%) in the OR group. There was no significant difference between the two groups. Similar results were observed in the instrument flushing fluid (Table 2).

**Table 2:** Result of tumour cells detected in the irrigation of peritoneum and instruments

Pre-operation	Post-operation	Lap (n=47)	Open (n=113)	P
+	+	3 (6.38%)	8 (7.07%)	0.87
+	—	15 (31.91%)	27 (23.89%)	0.29
—	+	2 (4.26%)	5 (4.42%)	0.96
—	—	27 (57.44%)	73 (64.60%)	0.39
Positive of instrument irrigation	48.51%	97.96	0.91	

The mean length of colon resected in the LCR group and OR group were (19.38±7.47) cm and (18.60±8.40) cm respectively. The distal margin to the rectal tumour were (4.19±2.52) cm and (4.16±2.00) cm and there was no positive margin in either group. Also, there was no difference between the two groups as far as the lymph node yield was concerned (LCR: 13.71±9.29; OR: 12.10±9.74, *P*=0.51). The rate of regional lymph node involvement was equivalent between both the groups. Although the number of the principle lymph nodes in laparoscopic group was higher than that obtained in the open group, the difference did not reach statistical significance (Table 3).

**Table 3:** Comparison of the Oncologic outcomes

		Lap	Open	*P*
GrossMorphology	Size of tumour (cm)	3.62±1.77	3.45±1.33	0.54
	Length of specimencm	14.54±5.55	16.63±5.92	0.15
	Length of coloncm	19.38±7.47	18.60±8.40	0.76
	Distal margincm	4.19±2.52	4.16±2.00	0.97
Clearance of lymph node	Total number	13.71±9.29	12.10±9.74	0.51
	Epicolic & paracolic LN	7.90±6.01(1.61)*	8.17±5.71(1.47)*	0.86
	Intermediate LN	3.06±2.96(0.42)*	2.77±4.02(0.23)*	0.74
	Principle LN	2.67±2.10(0.38)*	1.17±2.90(0.00)*	0.23

* In the bracket shows the mean number of involved lymph nodes

All the cases were available for follow-up for a median period of 13.8 months (range, 5-20 months). The local recurrence rate of the laparoscopic group was 2.13% (1/47), 1.77% (2/113) of the open group (*P*=0.88). The incidence of distant metastasis were 6.38% (3/47) and 6.19% (7/113) respectively (*P*=0.96).

## DISCUSSION

The advantages of laparoscopic surgery such as smaller incisions, reduced bleeding, quicker recovery and fewer complications have been well established. With the application of ultrasonic shears, endoscopic staplers and other instruments as also with maturing of the surgical skills minimally invasive surgery is being widely utilized in the treatment gastrointestinal cancers, particularly for the treatment of colorectal malignancy. However, laparoscopic curative colorectal resection has yet not gained widespread approval and most surgeons still view it with some caution due to concerns about its oncological feasibility and safety. Issues such as whether laparoscopic colorectal surgery for malignancy can conform to the oncological principles, whether the short-term outcomes and long-term survivals are comparable to open surgery and the problem of port-site metastases still remain controversial.

Since the first report of the tumour cells implantation at the port-site after laparoscopic surgery in 1993, this has become an important issue that hinders the application of laparoscopic surgery for treatment of colorectal cancer. The incidence of early port-site recurrence was 1.4%-21.0%. In the last 5 years, many experienced surgeons from have reported an incidence between 0-0.2%,^1^,^2^ which is not significantly different compared to that achieved by laparotomy. Thus it was concluded that the main reason for early reported port-site metastasis was the improper handling of the tumour causing dissemination of cells in the peritoneal cavity and seeding at the incision or port-site.

In 1993, we successfully undertook the first laparoscopic colorectal resection in our country. Since then, we have steadily gained more experience and standardized the operative procedure. In all or patients we strictly followed the “no-touch’ technique: avoiding direct manipulation of the tumour, limiting the instruments inserted and protecting the mini laparotomy with a plastic sleeve during the extraction of specimen. All these measures are suggested to prevent the implantation of tumour cells at port-sites. In our study, the incidence of tumour cells detected in the instrument flushing fluid in the two groups was not no significantly different. Similarly, the incidence of positive cytology rates in the peritoneal washing after operation were similar in both groups. We concluded that the laparoscopic surgery technique is unlikely to increase the risk of tumour cells dissemination in the abdominal cavity and the metastasis during the instrument exchanges. During the short-term of follow-up, no port-site metastasis or port-site or incision recurrence was observed in either group in our study.

Some other studies considered that the port-site implantation was caused by the so called “Chimney effect” of the pneumoperitoneum as presented by Tseng.^3^ This referred to the floating of the tumour cells in the abdominal cavity under the effect of CO_2_ pneumoperitoneum. During the operation with a reduction in the intraperitoneal pressure these cells could escape from the trocar’s outlets. Some of the tumour cells then adhered to the port-site or incision and the margins around them producing port-site implantation. There have been many reports challenging the hypothesis of the “Chimney Effect”. Iwanaka^4^ demonstrated that laparoscopic biopsy of tumour both under the condition of pneumoperitoneum and the gasless system. By comparing the port-site recurrence (PSR) rates between them, he concluded that CO_2_ pneumoperitoneum was not found to be essential for the development of PSR. Another animal experiment from Hao Wang^5^ showed that only under the consistently high pneumoperitoneum pressure and certain concentration of the tumour cells (1.6FNx01107/ml) port-site and visceral metastasis could develop. The experiment of Wittich^2^ also confirmed that the routine procedure undertaken during a laparoscopic operation could not result in development of port-site metastasis. The “Chimney Effect” did not occur in our experiment. None of the patients who had positive postoperative peritoneal washings subsequently develop port-site metastasis.

Besides the advantage of minimal invasion, laparoscopically performed colorectal resections can be oncological equally radical as compared to traditional operations. The laparoscopic procedure does not deviate from the steps of the traditional radical excision as it also includes the high ligation of the vessels, adequate length of the distal margin from tumour, adequate lymphadenectomy and mesenteric resection. The majority of clinical reports have confirmed these findings in terms of oncological radicality achieved by laparoscopic surgery for colorectal cancers as compared to laparotomy. Kockerling^6^ in a retrospective series of 116 patients with colon cancer undergoing laparoscopic resection reported adequate tumour resection. Franklin^7^ reported a randomized comparative study between 191 laparoscopic and 224 open colorectal resections for the malignant tumour. No significant differences were observed between the two groups in terms of the extent of lymph node harvest, the length of specimen, the distance of proximal or distal margin from the tumour.

In our series, all the pathological parameters including the length of bowel resection, distal margin from the inferior edge of the rectal tumour, the number of lymph node harvested and the positive rate for each station were not significantly different between the LCR and OR groups. Studies reported from overseas^8^,^9^ and our series indicate the extent of the lymph nodes harvest of laparoscopic operation is comparable to that after an open procedure. The short-term outcome shows the local recurrence rate and the survival between the two groups to be equivalent.

In conclusion, laparoscopic colorectal resection for the treatment of malignant cancer can be performed safely and effectively.
